# Home and community care services: a major opportunity for preventive health care

**DOI:** 10.1186/1471-2318-10-26

**Published:** 2010-05-22

**Authors:** Louisa R Jorm, Scott R Walter, Sanja Lujic, Julie E Byles, Hal L Kendig

**Affiliations:** 1 School of Medicine, University of Western Sydney, Locked Bag 1797, Penrith South DC NSW 1797, Australia; 2 The Sax Institute, PO Box 123, Broadway NSW 2007, Australia; 3 NSW Department of Health, LMB 961 North Sydney NSW 2059, Australia; 4 Research Centre for Gender, Health and Ageing, University of Newcastle, University Drive, Callaghan NSW 2308, Australia; 5 Ageing, Work and Health Research Unit, C/- CERA, Concord Hospital C25, Concord NSW 2139, Australia

## Abstract

**Background:**

In Australia, the Home and Community Care (HACC) program provides services in the community to frail elderly living at home and their carers. Surprisingly little is known about the health of people who use these services. In this study we sought to describe health-related factors associated with use of HACC services, and to identify potential opportunities for targeting preventive services to those at high risk.

**Methods:**

We obtained questionnaire data from the 45 and Up Study for 103,041 men and women aged 45 years and over, sampled from the general population of New South Wales, Australia in 2006-2007, and linked this with administrative data about HACC service use. We compared the characteristics of HACC clients and non-clients according to a range of variables from the 45 and Up Study questionnaire, and estimated crude and adjusted relative risks for HACC use with generalized linear models.

**Results:**

4,978 (4.8%) participants used HACC services in the year prior to completing the questionnaire. Increasing age, female sex, lower pre-tax household income, not having a partner, not being in paid work, Indigenous background and living in a regional or remote location were strongly associated with HACC use. Overseas-born people and those speaking languages other than English at home were significantly less likely to use HACC services. People who were underweight, obese, sedentary, who reported falling in the past year, who were current smokers, or who ate little fruit or vegetables were significantly more likely to use HACC services. HACC service use increased with decreasing levels of physical functioning, higher levels of psychological distress, and poorer self-ratings of health, eyesight and memory. HACC clients were more likely to report chronic health conditions, in particular diabetes, stroke, Parkinson's disease, anxiety and depression, cancer, heart attack or angina, blood clotting problems, asthma and osteoarthritis.

**Conclusions:**

HACC clients have high rates of modifiable lifestyle risk factors and health conditions that are amenable to primary and secondary prevention, presenting the potential for implementing preventive health care programs in the HACC service setting.

## Background

The Australian Home and Community Care (HACC) program provides services in the community to frail elderly and people with disabilities living at home and their carers [1]. It aims to support their independence, to enhance their quality of life and to prevent their inappropriate admission to long term residential care [1]. Services vary with the needs of the client and include nursing and allied health care, personal care, meals, household assistance, transport, day centres and respite care [1,2]. Lifestyle modification is not a major current focus of HACC service provision, but policymakers are beginning to recognise the health promotion role that the program can play. New service models emphasise the role of the HACC program in supporting gains in health and wellbeing [3].

HACC receives AUD1.8 billion annually from the Commonwealth and State governments supplemented by client fees that amount to 5% of revenue [4]. Around 3,300 agencies deliver HACC services to approximately 830,000 people annually [1]. HACC providers include local government, community and charitable groups, and private for profit organisations.

As the population ages, the demands for HACC services are likely to increase, along with demands for other health care services. Recent national reviews of the Australian health system [5-7] all highlight the need for better integration of pathways of care, and for greater emphasis on prevention of chronic conditions. Given the pivotal role of HACC services in caring for older people, they may provide an ideal opportunity for targeting preventive services to those with high prevalence of risk factors. However, little is known about the individuals who use these services, and there is scant evidence on which to assess the potential of community care as a setting for the delivery of preventive health interventions. HACC program data collections only report descriptive demographic data and indicators of service need [1,8]. The Australian national Survey of Disability, Ageing and Carers does not report specifically on HACC clients [9].

Two Australian studies--both more than a decade old, and both restricted to limited geographic areas--found that service use was independently related to older age, activity limitations and indicators of poor health (including self-rated health and health service use) [10,11]. A more recent study from Western Australia used HACC program data coupled with client assessments to show that the strongest predictors of HACC resource use were dependency in activities of daily living (ADL) or instrumental activities of daily living (IADL) and needs for clinical care [12].

These Australian findings were consistent with international studies, which have reported that impairments to ADL have the strongest association with use of community care; other important factors include being older, female, non-white, living alone, having fewer children, having multiple chronic conditions, and geographic variations in the availability of services [13,14].

The purpose of our study was to describe the health-related factors that are associated with use of HACC services in Australia in a large, contemporary, population-based sample. We used baseline questionnaire data from Australia's largest cohort study--the 45 and Up Study--linked with administrative data relating to HACC services.

## Methods

### The 45 and Up Study

The 45 and Up Study is a cohort study of more than 250,000 men and women aged 45 and over resident in New South Wales (NSW). Its methods are described in detail elsewhere [15]. Participants were randomly sampled from the Medicare Australia database and joined the study by completing a mailed self-administered questionnaire and providing consent for long term follow-up, including linkage to health records. The response rate was 18% [15]. Recruitment to the 45 and Up Study commenced in 2006 and was completed in 2008. Our study used data for the just over 100,000 participants who had joined the Study up to July 2008.

### Home and Community Care Program Minimum Data Set (HACC MDS)

The HACC MDS captures information on client characteristics, living arrangements, and HACC services received. It has around 75% coverage of HACC service providers and 85% coverage of HACC clients in NSW. This is because some smaller HACC providers do not contribute to the HACC MDS. HACC MDS data for individuals record the services that they receive on a quarterly basis. HACC MDS data for the period 1 July 2003 to 30 June 2008 were available for our study. These included data from two different versions of the HACC MDS: Version 1 (prior to April 2007) and Version 2 (April 2007 onwards).

### Linked data set

Linkage of 45 and Up Study and HACC MDS data was performed by the Centre for Health Record Linkage (CHeReL) [16]. The HACC MDS does not contain full name information; instead it includes a statistical linkage key based on five letters of name, date of birth and sex. The CHeReL used deterministic methods to link this key to personal identifiers from the 45 and Up Study.

For this study, we used only HACC MDS records that related to the quarter in which the participant completed the 45 and Up Study questionnaire, and each of the three previous quarters. We included all clients of HACC services in our analyses, including people who receive care for their own frailty or disability (care recipients) and their carers. It was not possible to readily distinguish these two user groups in HACC MDS version 2 data. Carers could be identified in HACC MDS version 1 data; a preliminary analysis using only this version found that 3.4% of HACC clients were carers only; excluding these participants had very little impact on effect estimates for associations with key predictor variables.

The analysis data set included questionnaire data for 103,041 persons and 16,371 linked HACC records relating to 4,978 persons.

### Data analysis

The characteristics of HACC clients and non-clients were compared according to a range of variables from the 45 and Up Study baseline questionnaire (available at http://www.45andUp.org.au). Psychological distress was measured using the Kessler-10 score [17] and functional capacity using the Medical Outcomes Study Physical Functioning scale [18]. Health conditions were classified according to the response to the question "Has a doctor ever told you that you have any of the following...". Remoteness of residence was assigned according to the mean Accessibility Remoteness Index of Australia Plus (ARIA+) [19] score for the postcode of residential address.

Crude and adjusted relative risks (RRs) for using HACC services were estimated using generalized linear models, specifying Poisson distribution with a robust error variance [20] To assess relationships with socio-demographic factors, independent of measures of 'need' for these services, we calculated RRs adjusted for sex, age, relationship status, income and physical functioning score. To explore associations between HACC use, lifestyle factors and health conditions, we calculated RRs adjusted for sex, age, relationship status and income. We did not adjust these for physical functioning score because of the causal relationships between lifestyle factors, ill-health and reduced functioning. To further investigate associations found for HACC use overall, we examined use of specific types of HACC service.

All analyses were carried out in SAS V9 [21]. All statistical tests were two-sided, using a significance level of p < 0.05.

### Ethical approval

This study was approved by the NSW Population and Health Services Ethics Committee, Cancer Institute NSW (reference 2008/05/077).

## Results

### Socio-demographic factors and HACC use

**Figure 1:**
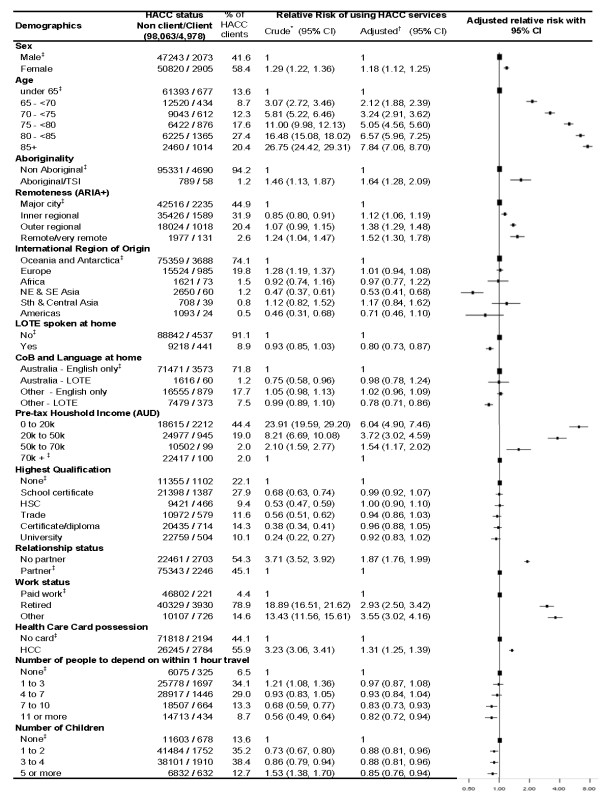
**Socio-demographic factors and use of Home and Community Care services**. TSI - Torres Strait Islander; LOTE - Language other than English; CoB - Country of birth The squares represent the relative risk estimates and the extending from the squares represent the 95% confidence intervals for the relative risks. Bigger squares represent the reference category. * Relative Risk of using HACC services. ^† ^Adjusted for age, sex, income, marital status and functional capacity. ^‡ ^Reference Category.

Of 103,041 45 and Up Study participants, 4,978 (4.8%) were clients of HACC services in the year prior to completing the questionnaire. Females were more likely to be HACC clients, and HACC use increased with age. People who had a partner were much less likely to use HACC services, and HACC use decreased with increasing household income (Figure 1).

After adjustment for age, sex, relationship status, income and level of physical functioning, level of educational attainment was not significantly associated with HACC service use. People who were not in paid work and those who were health care card holders were significantly more likely to be HACC clients (Figure 1). The likelihood of HACC use decreased slightly with participants' reported number of children, and with the number of people they could depend upon who lived within one hour's travel time.

The likelihood of HACC use increased with increasing geographic remoteness. Transport, domestic assistance, centre-based meals and a range of aids and equipment were more likely to be used by HACC clients who lived in rural and remote areas than those living in more accessible areas (data not shown).

People of Indigenous origin were more likely than others to be HACC clients. Indigenous HACC clients were more likely than other HACC clients to use home maintenance, centre-based nursing care and meals, social support, domestic assistance, transport and a range of medical care and other aids and equipment (data not shown).

People born overseas, especially those born in South East and North East Asia, and those who spoke a language other than English at home were significantly and substantially less likely to be HACC clients (Figure 1). HACC clients who spoke a language other than English at home were less likely than other HACC clients to use home-delivered meals and transport services and more likely to use centre-based day care (data not shown).

### Lifestyle factors and HACC use

**Figure 2:**
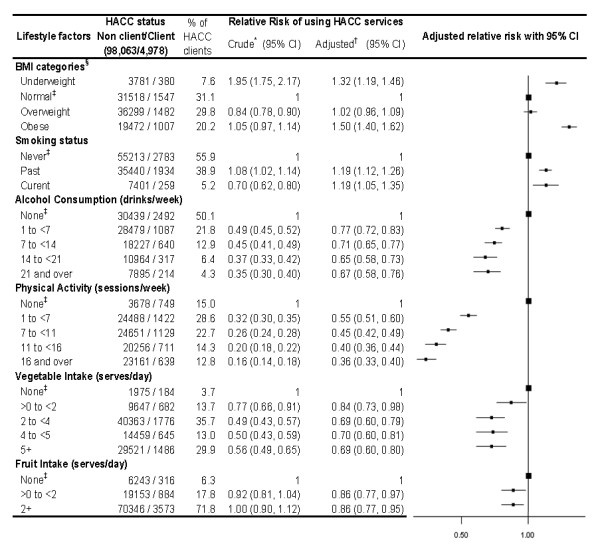
**Lifestyle factors and use of Home and Community Care services**. BMI - Body Mass Index. The squares represent the relative risk estimates and the extending from the squares represent the 95% confidence intervals for the relative risks. Bigger squares represent the reference category. * Relative Risk of using HACC services. ^† ^Adjusted for age, sex, income, marital status and functional capacity. ^‡ ^Reference Category. § Categories: Underweight (BMI<20), Normal weight (BMI 20-<25), Overweight (BMI 25-<30), Obese (BMI 30 and higher).

The adjusted results indicate that for a given sex, relationship status and income, the probability of using HACC services varied according to a range of lifestyle factors (Figure 2). The likelihood of being a HACC client was elevated both among people who were underweight and those who were obese. Level of physical activity had a very strong inverse relationship with HACC service use. The likelihood of HACC use was elevated for current and past smokers in comparison to never smokers, and decreased gradually with increasing levels of alcohol consumption, and increasing fruit and vegetable intake.

### Health status and HACC use

**Figure 3:**
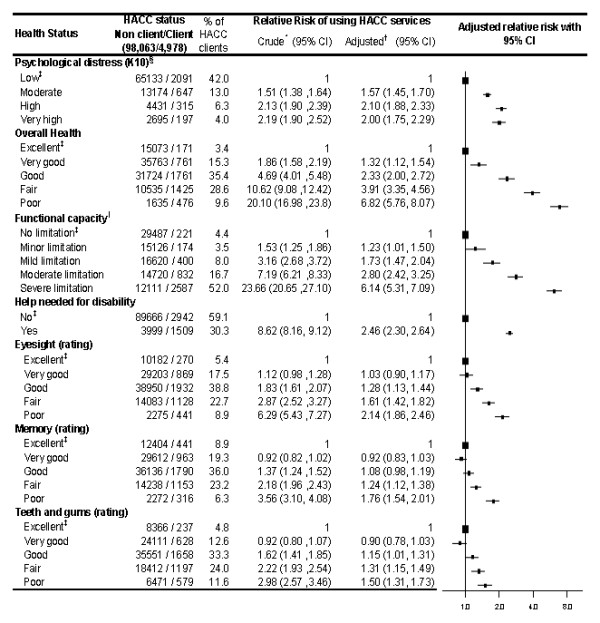
**Health status and use of Home and Community Care services**. The squares represent the relative risk estimates and the extending from the squares represent the 95% confidence intervals for the relative risks. Bigger squares represent the reference category. * Relative Risk of using HACC services. ^† ^Adjusted for age, sex, income, marital status and functional capacity. ^‡ ^Reference Category. ^§ ^Categories: low (score 10-15), moderate (16-21), high (22-29), very high (30 or higher). ^∥^Categories: No limitation (score of 100), minor limitation (95-99), mild limitation (85-94), moderate limitation (60-84), severe limitation (0-59).

People who used HACC services had worse health status than non-clients across a variety of indicators (Figure 3). The likelihood of HACC use was significantly elevated in people who had moderate, high or very high levels of psychological distress. Strong and substantive gradients of increasing HACC service use were observed with decreasing levels of physical functioning, and with declining self-ratings of overall health, eyesight, memory and teeth and gums. HACC clients were much more likely than other individuals to report that they regularly needed help with day to day tasks because of long-term illness or disability; however, only 30% of HACC clients reported this.

### Health conditions and HACC use

**Figure 4:**
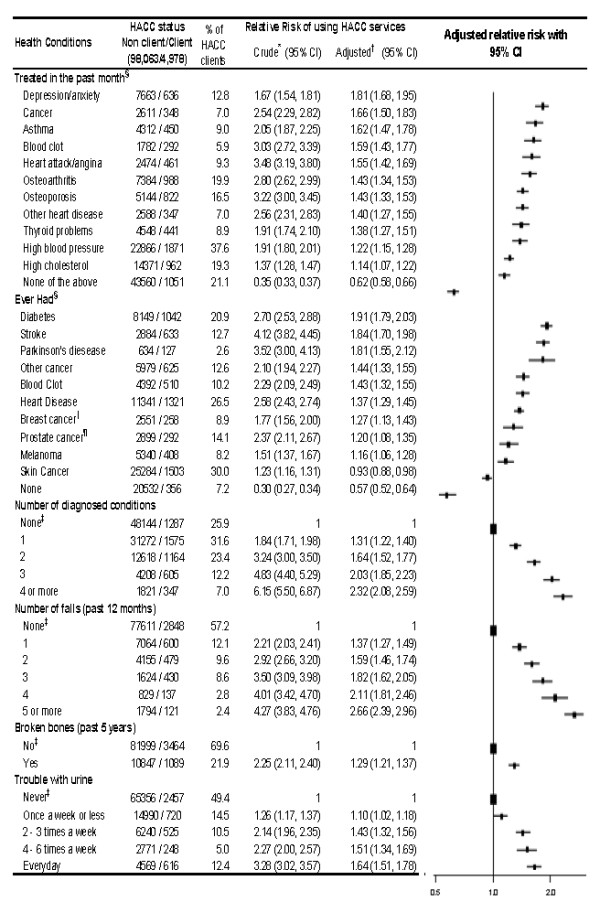
**Health conditions and use of Home and Community Care services**. The squares represent the relative risk estimates and the extending from the squares represent the 95% confidence intervals for the relative risks. Bigger squares represent the reference category. * Relative Risk of using HACC services. ^† ^Adjusted for age, sex, income and marital status. ^‡ ^Reference Category. ^§ ^Reference category = No. ^∥ ^Females only. ^¶ ^Males only.

The likelihood of HACC service use was elevated among people who reported a wide range of health conditions (Figure 4). There was a strong gradient of increasing HACC service use according to the reported number of falls in the past 12 months. There was a clear gradient of increasing likelihood of HACC use with increasing frequency of episodes of urinary incontinence.

Analysis by service type indicated that clients who reported diabetes were high users of centre-based care and centre-based allied health care, clients who reported stroke were high users of centre-based care and domestic assistance, and clients who reported fractures were high users of domestic assistance and personal care (data not shown).

## Discussion

We found that increasing age, female sex, lower pre-tax household income, not having a partner, not being in paid work, Indigenous background and living in a regional or remote location were key influences on use of HACC services. People who were born outside Australia or spoke a language other than English at home were significantly less likely to use these services. The higher use of HACC with lower income, after adjusting for other need-related variables, suggests that fees for HACC services are not a major barrier to their use.

Rates of HACC service use increased with increasing remoteness regardless of other need-related factors. This reflects targeting of services to special needs groups, as well as perhaps facilitation of access to services in rural areas through closer community networks [22] and variations in assessment practices [23]. Similar factors, as well as higher rates of disability, may contribute to higher rates of HACC service use among Indigenous people.

People born overseas and speaking a language other than English at home were around 20% less likely to use HACC services than other individuals. Studies in other Australian states have found that the proportion of HACC clients speaking a language other than English at home was similar to the population proportion [8,24], but they received fewer hours of service overall, and made less use of delivered meals and social activity groups [24]. Our findings regarding home-delivered meals were similar, but we found that HACC clients who spoke languages other than English at home were more likely than others to use centre-based day care, most likely reflecting the availability of culturally specific services in NSW.

Our study was the first to examine associations between a range of lifestyle-related factors and HACC service use. We found a U-shaped association with body weight: both underweight and obese individuals were significantly more likely to be HACC clients than people who were overweight or of normal bodyweight. Both these conditions reflect opportunities for improved nutrition among HACC clients. Underweight and malnutrition are particular problems among the elderly and are major contributors to hospitalisation and physical decline [25]. Consistent with our findings regarding bodyweight, eating no or few daily serves of fruits and vegetables was also more common among HACC clients, again identifying opportunity and needs for improving nutrition for this client group.

Rates of HACC service use were 2 to 3 times higher among people who were sedentary compared to other individuals. There was also a particularly strong association between HACC service use and number of falls in the past year; taken together these findings demonstrate the considerable potential for implementing programs to increase levels of physical activity, with a focus on improving strength and balance, in the HACC context. Good practice guidelines for such programs are already available [26].

Current smoking was also higher among HACC clients, indicating the potential to engage this client group in targeted smoking prevention programs. Conversely, HACC clients were more likely than other participants to abstain from drinking alcohol. This finding cannot be taken to indicate that alcohol consumption has beneficial effects, because it is possible that participants may have given up drinking as a result of poor health.

Consistent with earlier Australian studies [10-12], and as expected given that people receive HACC services because they have a disability, we found strong gradients of increasing HACC service use according to decreasing level of physical functioning, and poorer self-ratings of eyesight and memory. Reflecting the disabling nature of many chronic health conditions, we found also that HACC clients were more likely than non-clients to report having conditions including Parkinson's disease, stroke or diabetes, anxiety and depression, cancer, heart attack or angina, blood clotting problems, asthma and osteoarthritis. More detailed analysis to explore the service types used by these clients is required to assess the potential for implementing intensive chronic disease management programs through HACC services.

Important strengths of our study include its large population-based sample, the detailed data about socio-demographic, lifestyle and health-related factors that were available though the 45 and Up Study questionnaire, and the independent ascertainment of HACC service use through data linkage. It allowed us to characterise the health of HACC clients in a very comprehensive way.

It is possible that HACC clients in the 45 and Up Study were not representative of the broader HACC client population. In keeping with other similar large-scale population-based cohort studies, its response rate was 18% [15]. A comparative analysis found that the prevalence of many factors in the 45 and Up Study, including country of birth, educational attainment, fruit consumption, body-mass-index and falls, was similar to the NSW Population Health Survey (PHS), a population-based survey which has a response rate of around 60%. However, 45 and Up participants tended to have higher incomes, and had lower prevalence of smoking, high psychological distress, hypertension, diabetes and asthma [27]. This suggests that 45 and Up Study participants are in general "healthier" than the overall population. However, importantly, we have reported *relative *measures of effect (RRs) calculated from internal comparisons within the 45 and Study, which will be valid provided there is sufficient heterogeneity within the predictor variables [28]. Moreover, empirical data demonstrate that RRs for a wide range of exposure-outcome relationships in the 45 and Up Study are very similar to those calculated using 'representative' PHS data [27]. Any bias resulting from an absence from the 45 and Up Study of the sickest, most dependent, HACC clients would generally cause underestimation of the associations between health risk factors, health conditions and HACC use. Misclassification relating to the incomplete coverage of the HACC MDS is also likely to bias findings towards the null.

All of our predictor variables were based on self-report. In general, people tend to under-report lifestyle risk factors [29], and to under-report many but not all health conditions [30]. Again, any resulting bias would most often result in more conservative RR estimates in relation to HACC use.

As a cross-sectional study, ours could not identify temporal sequences of events, nor identify personal and service-related factors that can potentially prevent avoidable admissions of home care clients to hospital or residential aged care [31,32]. Longitudinal analyses needed to address questions of this type will become possible as the 45 and Up Study progresses.

## Conclusions

Our innovative linkage of the 45 and Up Study with HACC program data has shown that socio-demographic vulnerability (in particular, low income and not having a partner) and health needs are strong predictors of HACC service use. Further, modifiable lifestyle risk factors, and health conditions that are amenable to primary and secondary prevention (including underweight, obesity and falls), occur at a much higher rate among HACC clients than other individuals. Detailed analysis of how clients use specific services, and of client capacities and willingness to participate in intervention programs, will assist in developing and targeting preventive health interventions for delivery in the HACC service setting.

## Competing interests

The authors declare that they have no competing interests.

## Authors' contributions

LJ participated in the conception and design of the study, oversaw data analysis and drafted the manuscript. SRW performed the statistical analysis and helped to draft the manuscript. SL participated in the design of the study, supervised the statistical analysis and helped to draft the manuscript. JEB and HLK participated in the conception and design of the study and helped to draft the manuscript. All authors read and approved the final manuscript.

## Pre-publication history

The pre-publication history for this paper can be accessed here:

http://www.biomedcentral.com/1471-2318/10/26/prepub
